# COVID-19 vaccine hesitancy and attitudes of subjects with disability and their carers in Saudi Arabia: a cross-sectional study

**DOI:** 10.3389/fpubh.2024.1282581

**Published:** 2024-02-28

**Authors:** Anwar A. Sayed

**Affiliations:** Department of Basic Medical Sciences, College of Medicine, Taibah University, Madinah, Saudi Arabia

**Keywords:** attitudes, COVID-19, disability, health inequality, hesitancy, public health, Saudi Arabia, vaccines

## Abstract

The COVID-19 pandemic has caused significant disruption to countries worldwide, including Saudi Arabia. The fast preventative measures and the mass vaccine enrollment were vital to contain the pandemic in the country. However, vaccine hesitancy was a significant obstacle to taking the vaccine but was not previously explored. One hundred eighty-six subjects with disabilities were enrolled in this study in an attempt to explore their hesitancy and attitudes toward COVID-19 vaccines. Most participants were previously diagnosed with COVID-19 and had a close family who was also diagnosed with it. Most of them were willing to be vaccinated but had not received previous vaccinations. Official sources of information, e.g., TV/radio, were an essential factor driving their intention to get vaccinated. Beliefs that drove participants’ vaccine acceptance included vaccine safety, sufficient testing before its release, and its ability to protect from infection. The results of this seminal study provide insights to public health policymakers, which should be considered and taken together in light of other studies addressing the population’s vaccine hesitancy.

## 1 Introduction

The World Health Organization (WHO) defines disability as a result of the interaction between several health conditions and personal and environmental factors. The WHO estimates that about 16% of the global population experience a disability and live a shorter life span, a range of health inequalities, and a significantly higher risk of developing further comorbidities (1). In Saudi Arabia, disability is defined as a complete or partial deficiency, permanently, in the person’s capabilities, e.g., physical, sensory, or psychological. Examples of disability include but are not limited to visual impairment, mental disability, and learning difficulties (2). It is estimated that 7.1% of the Saudi population, i.e., over 1.4 million people, live with a disability, with over 830 thousand having a physical disability and over 800 thousand having a vision disability (3).

In March 2020, the WHO declared the novel coronavirus disease 2019 (COVID-19) a global pandemic (4). Since then, the world turned into turmoil, with countries doing their best to contain the infection. Saudi Arabia was one of the earliest countries to take stringent and progressive measures to tackle COVID-19 (5, 6). One of these measures was to enroll nationwide mass COVID-19 vaccination to everyone in the country, which seemed to be effective in tackling the virus’s spread and limiting its severe morbidity and mortality (7). However, vaccines are only effective when they are taken. As in many other countries, people in Saudi Arabia showed a demonstrable hesitancy and resistance toward COVID-19 vaccines (8–10). Such hesitancy toward the vaccines was not limited to themselves, i.e., people who want to receive the vaccines, but also their children (11).

Attitudes toward COVID-19 vaccination, including acceptance, hesitancy, and resistance, have been extensively studied in several populations in Saudi Arabia. However, not a single study addressed such an issue among this disadvantaged population, i.e., people with disability, who are at great risk health inequality, especially during COVID-19 (12). This study attempts to address this gap of knowledge by examining the attitudes of people with disability toward COVID-19 vaccination and assessing the possible contributing factors to such attitudes. The result of this study is expected to better inform public health policymakers to devise targeted strategies toward increasing vaccination uptake among subjects with disability.

## 2 Materials and methods

### 2.1 Study setting and participant recruitment

This study is a cross-sectional study in which a previously validated questionnaire (13) has been used. Briefly, the questionnaire is divided into four parts: participant characteristics, previous experience with vaccinations and COVID-19 vaccines, use and trust in different sources of information through a 4-point Likert scale, and finally attitudes and beliefs on COVID-19 vaccines through a 5-point Likert scale describing their agreement with the provided statements. All the questionnaire questions and the participants’ answers are described in the Tables 1–6.

**Table 1:** The ‘participants’ characteristics.

Participant characteristics	Number (*n* = 186)	Percentage
Gender	Male: 71	38.2%
	Female: 115	61.8%
Smoking status	Smoker: 24	12.9%
	Non-smoker: 162	87.1%
Age	35 (30–49) *	N/A
Level of education	Middle school: 3	1.6%
	High school: 27	14.5%
	Bachelor’s degree: 134	72.1%
	Postgraduate degree: 22	11.8%
Region of residence	Madinah: 84	45.1%
	Makkah: 65	34.9%
	Riyadh: 12	6.5%
	Eastern Province: 22	11.8%
	Qassim: 1	0.6%
	Al-Baha: 1	0.6%
	Jazan: 1	0.6%

^*^ Shapiro–wilk test was used to determine the distribution of the participants’ ages, which were expressed in median (interquartile range) as they were nonparametric. N/A, not applicable.

**Table 2:** Participants’ medical experience that could influence their intention to get vaccinated.

Participant characteristics	Number (*n* = 186)	Percentage
Personal medical history	Type I Diabetes: 34	18.3%
	Type II Diabetes: 87	46.8%
	Cardiovascular diseases: 15	8.1%
	Autoimmune disorders: 2	1.1%
	Obesity: 34	18.3%
	Healthy: 39	21%
Type of disability	Mobility disability: 102	54.9%
	Visual impairment: 60	32.2%
	Hearing impairment: 8	4.3%
	Speech and language disorders: 5	2.7%
	Mental disability: 2	1.1%
	Others: 9	4.8%
Previous vaccination (Hep B/Influenza)	Yes: 4	2.2%
	No: 182	97.8%

Hep B, Hepatitis B.

**Table 3:** Participants experience with COVID-19 infection.

Participant characteristics	Number (*n* = 186)	Percentage
Diagnosed with Covid-19 via a PCR test	Yes: 123	66.1%
	No: 63	33.9%
Immediate family/friends diagnosed with Covid-19 via a PCR test	Yes: 123	66.1%
	No: 63	33.9%
Hospital admission due to Covid-19	Yes: 35	18.8%
	No: 151	81.2%
Death of immediate family/friends due to Covid-19	Yes: 70	37.6%
	No: 116	62.4%

PCR, Polymerase Chain Reaction.

**Table 4:** Attitudes toward Covid-19 vaccination.

Participant characteristics	Number (*n* = 186)	Percentage
Would you consider taking the Covid-19 Vaccine for yourself?	Yes: 165	88.7%
	Maybe: 12	6.5%
	No: 9	4.8%
Would you consider taking the Covid-19 Vaccine for your immediate family/friends?	Yes: 173	93.0%
	Maybe: 7	3.8%
	No: 6	3.2%
If you were given a choice, which vaccine would you choose to take?	Pfizer/BioNTech: 131	70.4%
	AstraZeneca: 51	27.4%
	It does not matter/ no difference: 4	2.2%

**Table 5:** Use and trust in different sources of information.

The use of different sources of information			
Source of information used	Frequency of use	Percentage	*p* value
Social media	A lot: 55	29.6%	0.11
	To some extent: 67	36%	
	Very little: 46	24.7%	
	Not at all: 18	9.7%	
**TV and radio**	**A lot: 41**	**22.0%**	0.04*
	**To some extent: 65**	**34.9%**	
	**Very little: 51**	**27.4%**	
	**Not at all: 29**	**15.6%**	
Newspapers and magazines	A lot: 4	2.2%	0.25
	To some extent: 20	10.8%	
	Very little: 50	26.9%	
	Not at all: 112	60.2%	
**Friends and Family**	**A lot: 18**	**9.7%**	0.001**
	**To some extent: 62**	**33.3%**	
	**Very little: 70**	**37.6%**	
	**Not at all: 36**	**19.4%**	
Healthcare workers	A lot: 69	37.1%	0.1
	To some extent: 71	38.2%	
	Very little: 40	21.5%	
	Not at all: 6	3.2%	

The trust in the different sources of information			
Source of information trusted	Frequency of use	Percentage	*p* value
Social media	A lot: 22	11.8%	0. 13
	To some extent: 64	34.4%	
	Very little: 76	40.9%	
	Not at all: 24	12.9%	
TV and radio	A lot: 42	22.6%	0.94
	To some extent: 58	31.2%	
	Very little: 55	29.6%	
	Not at all: 31	16.7%	
Newspapers and magazines	A lot: 11	5.9%	0.14
	To some extent: 32	17.2%	
	Very little: 44	23.7%	
	Not at all: 100	53.8%	
Friends and family	A lot: 10	5.4%	0.24
	To some extent: 69	37.1%	
	Very little: 68	36.6%	
	Not at all: 39	21.0%	
Healthcare workers	A lot: 83	44.6%	0.56
	To some extent: 62	33.3%	
	Very little: 31	16.7%	
	Not at all: 10	5.4%	

Statements written in bold indicate statistical significance. A chi-square test was used.

* Denotes a *p* value < 0.05.

** Denotes a *p* value < 0.01.

**Table 6:** Concepts and beliefs about COVID-19 vaccines.

Statement	Level of agreement	Percentages	*p* value
**Covid-19 vaccine has been tested properly before its wide use in the vaccination program.**	**Strongly agree: 43**	**23.1%**	0.0001***
	**Agree: 52**	**28.0%**	
	**I do not know: 72**	**38.7%**	
	**Disagree: 10**	**5.4%**	
	**Strongly disagree: 9**	**4.8%**	
**Covid-19 vaccine is safe to use.**	**Strongly agree: 32**	**17.2%**	<0.00001****
	**Agree: 63**	**33.9%**	
	**I do not know: 75**	**40.3%**	
	**Disagree: 9**	**4.8%**	
	**Strongly disagree: 7**	**3.8%**	
**I believe that Covid-19 vaccine will protect me from getting infected.**	**Strongly agree: 26**	**14.0%**	0.005**
	**Agree: 55**	**29.6%**	
	**I do not know: 68**	**36.6%**	
	**Disagree: 25**	**13.4%**	
	**Strongly disagree: 12**	**6.5%**	
I believe that Covid-19 vaccine will protect me from having a severe Covid-19 infection.	Strongly agree: 47	25.3%	0.97
	Agree: 73	39.2%	
	I do not know: 51	27.4%	
	Disagree: 10	5.4%	
	Strongly disagree: 5	2.7%	
I recommend my family/friends to get vaccinated with Covid-19 vaccine.	Strongly agree: 70	37.6%	0.9
	Agree: 63	33.9%	
	I do not know: 40	21.5%	
	Disagree: 9	4.8%	
	Strongly disagree: 4	2.2%	
I believe that Covid-19 Vaccine is unsafe because it will alter/change my genes.	Strongly agree: 4	2.2%	0.46
	Agree: 13	7.0%	
	I do not know: 79	42.5%	
	Disagree: 37	19.9%	
	Strongly disagree: 53	28.5%	
I believe that Covid-19 Vaccine is unsafe because it will affect my fertility/getting pregnant.	Strongly agree: 1	0.5%	0.43
	Agree: 7	3.8%	
	I do not know: 80	43.0%	
	Disagree: 41	22.0%	
	Strongly disagree: 57	30.6%	
I believe that Covid-19 Vaccine is unsafe because we do not know its long-term side-effects.	Strongly agree: 19	10.2%	0.37
	Agree: 41	22.0%	
	I do not know: 68	36.6%	
	Disagree: 33	17.7%	
	Strongly disagree: 25	13.4%	
I do not need to get vaccinated as long as I am wearing a face mask and maintaining social distancing.	Strongly agree: 9	4.8%	0.88
	Agree: 18	9.7%	
	I do not know: 29	15.6%	
	Disagree: 71	38.2%	
	Strongly disagree: 59	31.7%	
I will only get vaccinated if it becomes mandatory, e.g., for Umrah or traveling.	Strongly agree: 27	14.5%	0.97
	Agree: 47	25.3%	
	I do not know: 12	6.5%	
	Disagree: 57	30.6%	
	Strongly disagree: 43	23.1%	
I worry that the vaccine will worsen my disability.	Strongly agree: 38	20.4%	0.08
	Agree: 34	18.3%	
	I do not know: 33	17.7%	
	Disagree: 40	21.5%	
	Strongly disagree: 41	22.0%	
The difficulty of reaching to vaccine clinics hinders my willingness to take it.	Strongly agree: 46	24.7%	0.39
	Agree: 37	19.9%	
	I do not know: 28	15.1%	
	Disagree: 32	17.2%	
	Strongly disagree: 43	23.1%	

Statements written in bold indicate statistical significance. A chi-square test was used.

** Denotes a *p* value < 0.01.

*** Denotes a *p* value < 0.001.

**** Denotes a *p* value < 0.0001.

The questionnaire was sent to subjects through social media, e.g., WhatsApp and Facebook in accordance with established guidelines for the use by health professionals and faculty staff (14). The population of Saudi Arabia is not entirely made of non-Saudi nationals, whose first language may not be Arabic, representing over 40% of the total population (15). Hence, Arabic and English versions were used to maximize the response rate among participants. The data collection of the study participants responses was conducted between May and September 2021.

The inclusion criteria of this study were subjects with disability, or their carers who can read and answer questions in either Arabic or English. No restriction was imposed on type of disabilities, such as mobility disability, visual impairment, hearing impairment, speech and language disorders and mental disability. The exclusion criteria of this study were subjects without disability, or those who cannot read or answer Arabic or English questionnaire. No restriction was imposed on the age of the participants.

The sample size was calculated using the open source OpenEpi, in which the sample size was estimated at 95% confidence level to be 108 participants (16).

### 2.2 Statistical analysis

The Shapiro–Wilk test was used to determine the distribution of numerical variables, e.g., participants’ age. Such determination was necessary to determine the appropriate parametric or nonparametric methods in case the variables were followed a normal or abnormal distribution, respectively. Descriptive quantitative methods were used to describe the collected variables, such as means and standard deviations (parametric data) and medians and interquartile ranges (nonparametric data), are calculated and presented using Microsoft Excel (Microsoft Corporation, Redmond, WA, USA). Chi-square test was used to determine the influence of categorical data on the participants’ willingness to vaccinate. Statistical significance was determined at a *p* value of less than 0.05.

### 2.3 Ethical considerations

All participants were debriefed on the study through a digital participant information sheet, and their participation was based on their consent. No personal or sensitive information was collected during the study. The Declaration of Helsinki was observed throughout the conduction of the study, which was approved by the Taibah University College of Medicine Research Ethics Committee approval number (TU-20-21) on April 2021.

## 3 Results

### 3.1 Participant characteristics

A total of 186 participants were included in this study as they completed the survey. The majority of respondents were female (*n* = 115, 61.82%), with over (*n* = 162, 87%) being non-smokers. The median participant age was 35 years old, with almost three quarters of them having a “bachelor’s degree (*n* = 134, 72.1%). Participants were from various regions, with just over (*n* = 84, 45%) being from Madinah, followed by Makkah (*n* = 65, 34.9%) and the Eastern province (*n* = 22, 11.8%). Detailed participants characteristics were described in Table 1.

Next, the study attempted to understand the participants past medical experience, their form of disability and whether they were previously vaccinated with other vaccines, such as Hepatitis B vaccines. Such an information may give us insights on their attitudes toward COVID-19 vaccines. Just less than half of the participants suffer from Type II diabetes Mellitus (*n* = 87, 46.8%), while just over one-fifth of the participants (*n* = 39, 21%) does not suffer from any comorbidities. Over half of the participants suffer from a mobility disability (*n* = 102, 54.9%), followed by less than one-third of participants with visual impairment (*n* = 60, 32.2%). Surprisingly, only 4 participants (*n* = 4, 2.2%) received previous vaccination in recent years. The detailed breakdown of the participants’ medical experience is shown in Table 2.

### 3.2 Participants experience with COVID-19 infection

In addition to the participants’ past medical history, it was important to understand their experience with COVID-19 and how badly it affected them and the people surrounding the study participants. Such an understanding will better inform us on their motives and drivers of their attitudes toward the vaccination.

Almost two-thirds of the participants had COVID-19 infection, with a positive polymerase chain reaction (PCR) test, as well as their immediate family and friends. Less than one fifth of the participants (*n* = 35, 18.8%) were hospitalized due to COVID-19 infection, and about 70 participants (*n* = 70, 37.6%) knew someone who died due to COVID-19 infection. The detailed breakdown of the participants’ experience with COVID-19 is shown in Table 3.

### 3.3 Attitudes toward COVID-19 vaccination and the impact of the use and trust of the different sources of information

Next, was to assess participants’ intentions toward COVID-19 vaccination and to identify whether there was a preference toward one vaccine over the other. The majority of the participants (*n* = 165, 88.7%) were willing to take the vaccine, and even more of them (*n* = 173, 93%) were willing to vaccinate their immediate family and friends. Interestingly, over 70% of the participants preferred to receive the Pfizer/BioNTech vaccine (*n* = 131, 70.4%) as compared to those who preferred AstraZeneca’s vaccine (*n* = 51, 27.4%) or those who had no preference to one vaccine over the other. The detailed breakdown of the participants’ intention is described in Table 4.

As previously described, the use of certain source of information does not necessarily equate to a trust in the reliability and validity of the information provided in that source of information (13). Therefore, it was important to assess the degree of use and trust in the different sources of information among the study participants, and analyzed to determine whether such use and trust affected the participants’ intention toward vaccination.

Healthcare workers were the most used and trusted sources of information, whereas newspapers and magazines were the least used and trusted sources of information. Interestingly, neither of the use and trust in these sources (healthcare workers and newspapers and magazines) significantly influenced the participants’ intention to vaccinate. The use of TV/radio and family and friends as sources of information were the only factors that significantly influenced participants’ willingness to vaccinate, as shown in Table 5.

### 3.4 Participants’ concepts and beliefs about COVID-19 vaccines

Given the participants’ access to the internet, social media and other sources of information, they were expected to be exposed to a number of misinformation regarding the COVID-19 vaccines. Moreover, such exposure could misinform the participants and make them adopt such ideas. Therefore, it was necessary to explore the participants’ concepts and beliefs about COVID-19 vaccines and examine whether such beliefs affected their intention to vaccinate.

Three statements were the main drivers that significantly influenced the participants to receive the COVID-19 vaccinations. These statements addressed the vaccine safety, being tested before its general enrollment, and its effectiveness in protecting the participants from getting infected. No other statements significantly influenced their decision to vaccination. Interestingly, it seemed that the participants still have doubts about the vaccine safety on their fertility/pregnancy and genes, with about (*n* = 80, 43%) and (*n* = 79, 42.5%) of the participants indicating such concerns, respectively. Furthermore, there was no consensus on the vaccine’s safety and whether it will worsen the subjects’ disability, nor on whether the vaccine accessibility would influence their decision to receive the COVID-19 vaccines, as described in Table 6.

## 4 Discussion

There has been a vast body of literature addressing the COVID-19 vaccine hesitancy among several sectors the Saudi population, e.g., healthcare workers (17, 18), pregnant and lactating women (19) and parents of young children (11, 20). Such hesitancy was essential to critically assess especially in such times of mass information and misinformation. However, none of these studies addressed COVID-19 vaccine hesitancy among subjects with a disability. This population is estimated to exceed 1.4 million subjects. Furthermore, subjects with disabilities usually face health inequalities, i.e., accessing routine healthcare such as cancer screening (21), and are likely to face similar issues in the context of COVID-19 (22).

The high acceptability and willingness to vaccinate with COVID-19 among the participants of this study is not surprising. Previous reports about vaccine hesitancy in Saudi Arabia has demonstrated high percentages of participants willing to be vaccinated, such as in the studies by Al-Zalfawi et al. (23) and Alfageeh and colleagues (24). Such acceptance may be attributed to the fact that the majority of the Saudi population has already been vaccinated, without any noticeable major adverse reaction. As of May 2023, over 68.5 million vaccination doses have been given in Saudi Arabia (25).

Social media platforms, such as Facebook and X (formerly known as Twitter), have been instrumental in shaping public attitudes toward vaccination. Skafle and her team reviewed over 700 articles in which they found that social media were a major contributing factor in shaping people’s opinion about vaccines. The misinformation on social media hindered people’s willingness to vaccinate with COVID-19 vaccines (26). Similarly, in Saudi Arabia, Al Naam and colleagues also highlighted the importance of the source of information and that social media led to lower vaccine acceptability than those who used official sources of information (27). On the other hand, the results of this study did not show an effect for the use of social media as almost half of the participants used social media, yet it was not an influencing factor on their intention to vaccinate. Such discrepancy may be attributed to the assumption used in other studies that using social media inherently means trust in them by users. However, in this study, the study tool allowed the differentiation between the use and trust in the different sources of information, including social media. It was clear these two concepts, use and trust, are not necessarily concordant.

Furthermore, over 77% of the participants in this study trusted healthcare workers as a reliable source of information, confirming the previous findings by Bagalb and colleagues that doctors recommendation was the most influencing factor behind vaccine acceptance (19). Similarly, using official sources of information, e.g., TV and radio, let people toward vaccination, in line with the results of this study (28).

Whether short or long term, the uncertainty toward the possible adverse reactions has been a significant concern and driver of vaccine hesitancy. In this study, almost half of the participants were not sure about the long-term safety of the vaccines, the possibility of it affecting the participants’ genes and its safety in pregnancy. Such concerns are not unheard of and have been previously reported. De Brabandere et al. (29) showed in their systematic review that safety concern, among pregnant women, was the most important factor driving vaccine hesitancy, as was in Saudi Arabia too (19). Temsah and colleagues also reported the concern of long-term effect of the vaccine as a driver of vaccine hesitancy in Saudi Arabia (28), while the work of Aldossari et al. (30) highlighted the concern of genetic abnormalities as a result of the vaccine. Healthcare providers and public health policymakers should actively address such uncertainty by delivering targeted educational campaigns in order to educate subjects with disability.

Unfortunately, the body of literature addressing COVID-19 vaccine hesitancy is very limited. Only two original articles were retrieved (31, 32), and three short communication articles (33–35) addressed COVID-19 vaccine hesitancy among subjects with disabilities in the United States and the United Kingdom. The participants of this study demonstrated concerns about vaccine safety, which aligns with the results of all these studies (31–35). This study, being the first of its kind in Saudi Arabia, addresses a gap in the literature. This study showed a link between the use of particular sources of information and its impact on their decision to vaccinate. This was in line with the work of Umucu and colleagues who indicated that access to a reliable source of information contributes to COVID-19 vaccine hesitancy among subjects with disabilities (32).

Saudi Arabia was one of the countries that enforced progressive public measures, including social distancing measures (5, 6). It was interesting to assess whether participants believed such measures to enough to protect them from the infection and suffice instead of the vaccine. The majority of participants in this study did not agree to such belief, in contrast to the finding of Almojaibel and his team, in which most of their respondents reported such a belief (36). While it is true that these measures were effective in reducing COVID-19 morbidity and mortality in Saudi Arabia in the earlier phases of the pandemic (7), vaccination was still important and could not be overlooked.

Although every effort was made to ensure excellent execution of the study, some limitations could be noted. This is a retrospective cross-sectional study, which in essence is fit for purpose as a population-based survey and being cost-effective. However, such types of study are difficult to derive definitive cause/effect factors. Furthermore, such studies could also carry the risk of sampling bias (37). Hence, interpreting such studies should be carefully considered and taken in light of other studies. Another notable consideration is the limited number of participants in this study, despite efforts to maximize participation in this study. Although the number of participants was low compared to other studies in Saudi Arabia, it is important to note that this was the first study addressing participants with disabilities. Hence, there is no valid reference to the participation of subjects with disabilities in such studies. Furthermore, those with a visual or intellectual disability who could not read or understand the questionnaire may not be able to respond to this questionnaire. Hence, this study may not represent their attitudes toward COVID-19 vaccination. Lastly, the study did not assess subjects with no history of vaccination. However, it is pretty unlikely for a person in Saudi Arabia to have no immunization history unless they live in a distant rural area with no access to healthcare or educational facilities. This is because Saudi Arabia has a national immunization program covering a wide range of infectious diseases, a prerequisite to joining school. Additionally, Saudi Arabia has imposed a COVID-19 mandate for the first two doses, which is required to enter any enclosed space, e.g., buildings. Nevertheless, these limitations remain as the perspectives of those with no vaccination history are crucial for understanding the broader spectrum of vaccine attitudes and behaviors.

The result of this study further clarifies the attitudes and factors influencing vaccine hesitancy among subjects with disabilities in Saudi Arabia. The study showed key similarities between subjects with disabilities and the public, e.g., the use of social media and their concerns regarding vaccine safety and potential adverse reactions. Healthcare policymakers could leverage such critical information to deliver targeted messages and address the concerns of subjects with disabilities through different information media, including social media platforms.

## 5 Conclusion

The mass COVID-19 vaccination programs in Saudi Arabia were vital in containing the infection, however, they were also associated with vaccine hesitancy and resistance among the public. Understanding the factors that influence such hesitancy are key to public health policymakers. Knowing and addressing these factors will allow us to provide timely scientific recommendations to the public, enhancing their acceptance significantly (38). Afterall, the vaccine is only effective when people take it, and such hesitancy may hinder the official efforts to curb the infection.

## Data availability statement

The raw data supporting the conclusions of this article will be made available by the author, without undue reservation.

## Ethics statement

The studies involving humans were approved by Taibah University College of Medicine Research Ethics Committee. The studies were conducted in accordance with the local legislation and institutional requirements. The participants provided their written informed consent to participate in this study.

## Author contributions

AS: Conceptualization, Data curation, Formal analysis, Funding acquisition, Investigation, Methodology, Project administration, Resources, Software, Supervision, Validation, Visualization, Writing – original draft, Writing – review & editing.
